# Is it a supplementary benefit to use anti-inflammatory agents in the treatment of type 2 diabetes?

**DOI:** 10.1186/s13104-017-2785-4

**Published:** 2017-09-08

**Authors:** Nzogang K. Patrice, Donkeng J. Martial, Telefo P. Bruno

**Affiliations:** 1 Department of Biochemistry, University of Dschang and District Hospital of Batcham (West Cameroon), Dschang, Cameroon; 2 Department of Biochemistry, University of Dschang and District Hospital of Tibati (Adamaoua Cameroon), Dschang, Cameroon; 3 Department of Biochemistry, University of Dschang, Dschang, Cameroon

**Keywords:** Type 2 diabetes, Inflammation, hs-CRP, Glycated haemoglobin and arterial pressure

## Abstract

**Objective:**

The aim of this study was to investigate an independent correlation between high sensitivity C-reactive protein (hs-CRP) and glycated haemoglobin (HbA1c) on one side and between hs-CRP and arterial pressure in well glucose controlled type 2 diabetics on the other side.

**Results:**

The mean of HbA1c was 6.37% in this study and 70.10% of participants had an HbA1c less than 7%. A positive correlation between hs-CRP and HbA1c was found in the current study (r = 0.232; P = 0.043) and we observed a decrease of 0.79% of HbA1c of the participants when their hs-CRP was less than 1 mg/l compared to that of the participants who had hs-CRP more than 1 mg/l (5.75 ± 1.25% VS 6.54 ± 1.42% *P* value = 0.04 Student). No correlation was found between hs-CRP and arterial pressure in this study. These results could justify the perspective use of anti-inflammatory drugs in the management of T2D. However the presence of participants with HbA1c levels greater than 7% makes plausible the influence of confounding factors on the observed correlations.

**Electronic supplementary material:**

The online version of this article (doi:10.1186/s13104-017-2785-4) contains supplementary material, which is available to authorized users.

## Introduction

Obesity and associated metabolic diseases are some major public health problems. This is justified because of their prevalence and the microvascular and macrovascular complications related [1]. Type 2 diabetes (T2D) is one of these metabolic diseases and its prevalence in the African region is 4.9% [2]. In Cameroon specifically, the prevalence is 3.3% according to a study conducted at the Biyem-Assi district hospital [3]. Taking into consideration the actual estimations, this prevalence might increase in the years to come [4, 5]. Therefore the description of pathophysiological mechanisms of this disease becomes not only an intellectual exigency but also a veritable urgency. Some evidences highlight a possible key role played by a chronic low grade inflammation in the development of insulin resistance (IR) and insulin deficiency (ID) observed in the T2D [1], [6–8]. This hypothesis is supported by the discovery or rediscovery of the hypoglycaemic effects of the anti-inflammatory drugs [7, 9–11]. However, if many studies explore the link between inflammation and T2D in the literature, there aren’t many studies that give clear answer to if it is judicious to use anti-inflammatory drugs in the management of T2D? The aim of this study was to investigate an independent correlation between high sensitivity C-reactive protein (hs-CRP) and glycated haemoglobin (HbA1c) on one side and between hs-CRP and arterial pressure in well glycaemic controlled type 2 diabetics on the other side.

## Main text

### Methods

#### Study population

We conducted a cross sectional study over a period of 2 months from 1st November to 31st December 2016. This study was done at the district hospital of Dschang which is a third category hospital in the west region of Cameroon. We included in this study all patients known to be type 2 diabetic since at least 3 months and well glycaemic controlled at the moment of inclusion in the study. Well glycaemic controlled type 2 diabetic was defined in this study as the diabetic who had a fasting blood sugar less than 1.30 g/l and a post prandial blood sugar less than 1.80 g/l during the last 3 months which preceded study inclusion. Were excluded: all patients who had been treated for infectious disease during the last 4 months preceding study inclusion, pregnant women, tobacco consumers, patients on anti-inflammatory drugs and/or on lipid lowering agents and patients who didn’t respect their treatment or with an acute or chronic T2D complication. The sample size was 72 participants obtained using Lorentz’s formula, with: as global T2D prevalence 4.9% [2], confidence level at 1.96 and error margin 0.05.

#### Setting

We obtained research authorisation from the administration of the Dschang district hospital. Biochemical dosages were done in the biochemical laboratory of the said hospital. HbA1c concentrations were determined by the ions exchange resin using fresh blood of participants collected in EDTA tubes. DIALAB^®^ laboratory reagents were used for this dosages. For hs-CRP, the method used was turbidimetry with the reagents of the Prestige Diagnostics^®^ laboratory and blood was also collected in EDTA tube. Blood samples were collected from fasting patients in the morning precisely between seven and twelve AM. For these dosages, spectrophotometer BioSystems^®^ BTS-310 was used. Arterial pressure considered for each participant in this study was the mean of the three arterial pressures scaled at its past three consultations.

#### Statistical analysis

Data was analysed using the software Epi info version 3.5.4, SPSS version 20 and Microsoft Excel 2013. Correlation between quantitative variables was explored by Pearson’s correlation coefficient and these correlations were also explored graphically. The statistical tests used in this study were: ANOVA to compare variances, Student test when the variances were equal to compare the means and to investigate whether Pearson’s correlation coefficient was significant and Kruskal–Wallis (Mann–Whitney) tests when the variances were unequal also to compare the means and the Fisher’s exact test to compare the proportions. All these tests were two-sided and they were statistically significant for a P-value less than 5%. Confidence interval in this study was 95%.

### Results

Seventy-seven participants were enrolled. The mean age ± SD was 61.09 ± 12.43 years with extremes of 29 and 83 years. Additional file 1 shows the sociodemographic features of the study population. The majority of our study population observed the dietetics measures and physical activities prescribed in the treatment of T2D as shown on Additional files 2 and 3. The median of diabetes duration was 04 years with an interquartile interval of 01–08 years. Regarding the clinical features of participants, 72.70% were obese and 70.10% were patients with well-controlled diabetes based on HbA1c levels (Additional file 4). The mean ± SD of body mass index (BMI) was 27.90 ± 4.03 kg/m^2^. Globally, obesity was more marked in women compared to men (Additional file 5).

The clinical, sociodemographic and therapeutic features of the participants didn’t influence their means of hs-CRP and HbA1c. However the mean of hs-CRP of the patients with well-controlled diabetes based on HbA1c levels was significantly less than that of the patients with non-controlled diabetes (Additional files 6 and 7). BMI and waist measurement had no correlation with hs-CRP (Additional files 8, 9). A negative correlation was found between hs-CRP and daily doses of insulin used by patients on insulin therapy (r = −0.908; P = 0.012) (Additional file 10). Oral glucose control agents also showed an anti-inflammatory effect but it wasn’t significant (Additional file 11).

**Fig. 1:**
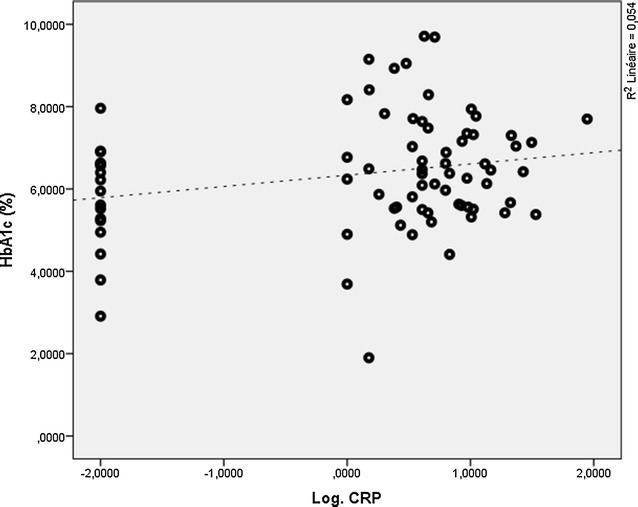
Dispersion of HbA1c and hs-CRP in the study population

**Fig. 2:**
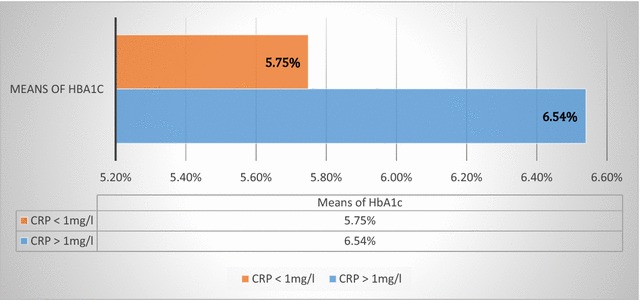
Comparing between the mean of HbA1c in two group formed from the study population on the base of hs-CRP concentration

The mean of HbA1c ± SD was 6.37 ± 1.41% with the extremes being at 1.90% and 9.71%. We observed a positive correlation between hs-CRP and HbA1c (r = 0.232; P = 0.043) as detailed on Fig. 1. Furthermore, when we compare the means of HbA1c of the participants who had hs-CRP less than 1 mg/l to that of participants who had hs-CRP levels greater than 1 mg/l, we have a significant decrease of 0.79% in the first group (5.75 ± 1.25% VS 6.54 ± 1.42% P-value = 0.04 Student) (Fig. 2). Nevertheless, when we observe the relation between HbA1c and hs-CRP in the well-controlled and non-controlled diabetes groups separately, the correlations are not significant in the two groups (Additional file 12). But, even if these correlations are not significant, tendency are queerly positive in well-controlled diabetes and negative in non-controlled diabetes. No significant correlation was noted between hs-CRP and arterial pressure as seen in Additional files 13 and 14.

### Discussion

We conducted a cross sectional study which aimed to analysing the relation between hs-CRP, HbA1c and arterial pressure. With this purpose, poor glycaemic controlled participants had to be excluded in order to minimise the effect of confounding factors on HbA1c and arterial pressure. We did the same for participants who presented factors that could influence hs-CRP.

Clinical and sociodemographic features of the participants enrolled in this study was similar to the characteristics of type 2 diabetics described in other studies [6, 12]. These participants in their majority respected the hygienic and dietetic measures. Moreover, the drugs taken corresponded to those prescribed in the treatment of T2D [4, 13]. More than two-third of the participants were controlled (70.10%) with an HbA1c less than 7%. No significant differences were noted between the clinical, sociodemographic and therapeutic features of well-controlled and non-controlled diabetes patients that could explain the non-controlled diabetes status. This finding was surprising because it’s known that physical activity and nutrition practice have some effects on plasma level of hs-CRP and HbA1c [14–17]. But the small number of participants who didn’t perfectly respect the hygienic and dietetic measures could explain our result.

The observation of a global obesity more marked on women (29.02 ± 4.14 kg/m^2^ vs. 27.15 ± 3.12 kg/m^2^ P-value = 0.04) as well as android obesity (100.29 ± 11.32 cm vs. 98.21 ± 12.22 cm), let us to suppose that hs-CRP in this study would be significantly higher in women than in men, given the theoretic link between obesity and chronic low grade inflammation in T2D. But this was not the case. High sensitivity CRP wasn’t equally associated with age and diabetes duration all known as poor glycaemic control factors [12]. This result differs from those of Wener et al. and Woloshin et al. who found a positive correlation between hs-CRP, female sex and age [18, 19]. However, these studies were done on non-diabetic population with study samples greater than ours. Tutuncu et al. also showed a positive correlation between hs-CRP and female sex in a study done on 21.485 type 2 diabetic participants [20]. These methodologic distinctions, especially the difference between our sample sizes could explain the difference observed in our results. When we look at the direct influence of obesity on the variation of hs-CRP in this study, the association remains non-significant. Indeed, we found no correlation between body mass index and hs-CRP (r = 0.053; not significant) as well as waist measurement (r = 0.046; not significant). This differs from the observations of Forouhi et al. and Ford who showed that, hs-CRP is associated to body mass index and waist measurement [21, 22]. Also in these studies, there are some methodologic differences with ours that could explain this contradictory result. In fact, in the study of Forouhi et al. study sample included only non-diabetic people while that of Ford included both diabetic and non-diabetic people and the sample size was higher than ours. But, observation of an association between hs-CRP, body mass index and waist measurement at the same time in Europeans and South Asians in the study of Forouhi et al. where sample study involved only 57 Europeans and 56 South Asians suggest that the fundamental methodologic difference between our studies is the sample constitution not the sample size. Our result therefore highlights so paradoxically a negative influence of diabetic state on the plasmatic CRP concentration. But, this could have all its meaning if we take into consideration the already known anti-inflammatory effect of Oral Glucose Control Agents and insulin [23–26] treatment on which all our participants were. This anti-inflammatory effect of insulin was highlighted in this study when we found a strong negative correlation between daily doses of insulin of the participants on insulin-therapy and hs-CRP (r = −0.908; P = 0.012). This result is similar to that observed in the literature [23, 27, 28].

We found a positive correlation between hs-CRP and HbA1c (r = 0.232; P = 0.043). This result is comparable to that of Bahceci et al. Sarinnapakorn et al. also found this positive correlation, and just as us they didn’t find a correlation between hs-CRP, arterial pressure, age and body mass index [29, 30]. It should be noted that in our study, this positive correlation between hs-CRP and HbA1c was highlighted in the majority (70.10%) of well glycaemic controlled participants based on HbA1c levels. These arguments together with the fact that we observed a decrease of 0.79% of HbA1c when hs-CRP is less than 1 mg/l let us to believe that an anti-inflammatory therapy could have a benefit in the treatment of T2D. However, when we separately compare the evolution of hs-CRP and HbA1c in the well-controlled diabetes patients group and in the non-controlled diabetes patients group, correlation between HbA1c and hs-CRP become non-significant probably because of our sample size. But, tendencies between these biochemical markers are strangely positive in the well-controlled diabetes patients and negative in the non-controlled diabetes patients. This fact let us suggest that, the link between hs-CRP and HbA1c in T2D could be non-linear with a horizontal asymptote. To explain this observation, we presupposed that it could be in link with the anti-inflammatory effect of oral glucose control agents and insulin. But, the means of the daily doses of these treatments weren’t greater in the non-controlled diabetes patients compared to that of well-controlled diabetes patients (Table 1). Even if this observation wasn’t significant, it could be interesting to confirm or nullify it in future studies.

**Table 1:** Comparing between the mean of daily doses of antidiabetic agents in well-controlled diabetes patients and that of non-controlled diabetes patients

Medicines	Well-controlled T2D	Non-controlled T2D	P value
Metformin (g/day)	1.81 ± 0.43 (50)	1.92 ± 0.23 (21)	0.70
Glibenclamide (mg/day)	55.00 ± 6.51 (05)	7.00 ± 2.12 (02)	0.18
Glimepiride (mg/day)	3.00 ± 0.97 (22)	2.30 ± 0.48 (10)	0.00*
Insulin (IU/day)	25.50 ± 11.70 (04)	43.00 ± 4.24 (02)	0.12

The values which we have represented in this table represent for each medicine the mean ± SD of the daily doses used in each group

Asterisk indicate significance

(): Number of observation

## Limitations

Though our study showed the correlation between HbA1c and hs-CRP on well glucose controlled participants, the presence in the study sample of 29.90% of non-controlled diabetics according to HbA1c criteria constitute a limit. This fact brings about a doubt on the independence of the correlations observed in this study.

## Additional files

**Additional file 1.** Sociodemographic features of the study population.

**Additional file 2.** Distribution of participants in relation to their feeding practices.

**Additional file 3.** Distribution of participants in relation to their physical activity.

**Additional file 4.** Clinical features of participants.

**Additional file 5.** Means of body mass index (BMI) and waist measurement in relation to sex.

**Additional file 6.** Means of hs-CRP and HbA1c in relation to the sociodemographic and clinical features of participants.

**Additional file 7.** Means of hs-CRP and HbA1c in relation to feeding practices and physical activity level of participants.

**Additional file 8.** Dispersion of BMI and hs-CRP in the study population.

**Additional file 9.** Dispersion of waist measurement and hs-CRP in the study population.

**Additional file 10.** Dispersion of the daily doses of insulin used by patients on insulin-therapy and hs-CRP.

**Additional file 11.** Dispersion of the daily doses of oral glucose control agents used by the participants and hs-CRP.

**Additional file 12.** Dispersion of HbA1c and hs-CRP in well-controlled diabetes patients and in non-controlled diabetes patients.

**Additional file 13.** Dispersion of diastolic arterial pressure and hs-CRP in the study population.

**Additional file 14.** Dispersion of systolic arterial pressure and hs-CRP in the study population.

## Abbreviations

CRERSHC: Comité Régional d’Ethique de la Recherche en Santé Humaine du Centre
HbA1c: glycated haemoglobin
hs-CRP: high sensitivity C-reactive protein
IR: insulin resistance
ID: insulin deficiency
T2D: type 2 diabetes
