# Supplementary material for: Is it a supplementary benefit to use anti-inflammatory agents in the treatment of type 2 diabetes?
Source: BMC Res Notes. 2017 Sep 8;10:471. doi: 10.1186/s13104-017-2785-4 (PMC5591512; doi:10.1186/s13104-017-2785-4)
Supplement: Supplementary file 1 — Additional file 1. Sociodemographic features of the study population. [file 13104_2017_2785_MOESM1_ESM.pdf]

Table S1: Sociodemographic features of the study population

	Frequency	Proportions (%)	CI at 95%
**Sex** (n=77)			
Male	46	59.70	47.90% - 70.80%
Female	31	40.30	29.20% - 52.10%
**Age groups** (n=77)			
Less than 50 years	15	19.50%	11.30% - 30.10%
More than 50 years	62	80.50%	69.90% - 88.70%
**Marital situation** (n=77)			
Married	67	87.00	77.40% - 93.60%
Unmarried	2	2.60	0.30% - 9.10%
Widower	8	10.40	4.60% - 19.40%
**Origin Region of participants** (n=77)			
West region of Cameroon	73	94.80	87.20% - 98.60%
North-west region of Cameroon	2	2.60	0.30% - 9.10%
Centre region of Cameroon	1	1.30	0.00% - 7.00%
Littoral region of Cameroon	1	1.30	0.00% - 7.00%
