# Supplementary material for: Is it a supplementary benefit to use anti-inflammatory agents in the treatment of type 2 diabetes?
Source: BMC Res Notes. 2017 Sep 8;10:471. doi: 10.1186/s13104-017-2785-4 (PMC5591512; doi:10.1186/s13104-017-2785-4)
Supplement: Supplementary file 2 — Additional file 2. Distribution of participants in relation to their feeding practices. [file 13104_2017_2785_MOESM2_ESM.pdf]

Table S2: Distribution of participants in relation to their feeding practices

	Frequency	Proportions (%)	CI at 95%
**Meal number per day** (n=77)			
Two meals	12	15.60	8.30% - 25.60%
Three meals	61	79.20	68.50% - 87.60%
Four meals	2	2.60	0.30% - 9.10%
More than four meals	2	2.60	0.30% - 9.10%
**Meals frequency** (n=77)			
Morn, noon and afternoon	63	81.80	71.40% - 89.70%
Late in the night	2	2.60	0.30% - 9.10%
**Gnaw between meals (n=77)**	36	46.80	35.30% - 58.50%
**Juice consumption (n=77)**	2	2.60	0.30% - 9.10%
**Alcohol consumption (n=77)**	31	40.30	29.20% - 52.10%
