# Supplementary material for: Is it a supplementary benefit to use anti-inflammatory agents in the treatment of type 2 diabetes?
Source: BMC Res Notes. 2017 Sep 8;10:471. doi: 10.1186/s13104-017-2785-4 (PMC5591512; doi:10.1186/s13104-017-2785-4)
Supplement: Supplementary file 3 — Additional file 3. Distribution of participants in relation to their physical activity. [file 13104_2017_2785_MOESM3_ESM.pdf]

Table S3: Distribution of participants in relation to their physical activity

	Frequency	Proportions (%)	CI at 95%
**Regular physical activity** (n=77)	27	35.10	24.50% - 46.80%
**Number of session per week** (n=27)			
Less than three	16	59.30	38.80% - 77.60%
Three	6	22.20	8.60% - 42.30%
More than three	5	18.50	6.30% - 38.10%
**Time of each session** (n=27)			
30 min	4	14.80	4.20% - 33.70%
More than 30 min	23	85.20	66.30% - 95.80%
**Global physical activity** * (n=77)			
Weak	40	51.90	40.30% - 63.50%
Moderate	16	20.80	12.40% - 31.50%
Intense	2	2.60	0.30% - 9.10%
None	19	24.70	15.60% - 35.80%

* Takes into consideration the transport mode used to go to work, the duration of walking per week, the type of work performed.
