# Supplementary material for: Is it a supplementary benefit to use anti-inflammatory agents in the treatment of type 2 diabetes?
Source: BMC Res Notes. 2017 Sep 8;10:471. doi: 10.1186/s13104-017-2785-4 (PMC5591512; doi:10.1186/s13104-017-2785-4)
Supplement: Supplementary file 4 — Additional file 4. Clinical features of participants. [file 13104_2017_2785_MOESM4_ESM.pdf]

**Table S4**: Clinical features of participants

| | Frequency | Proportions (%) | CI at 95% |
|---|---|---|---|
| **Familial past history of T2D*** (n=77) | | | |
| Yes | 37 | 48.10 | 36.50% - 59.70% |
| No | 40 | 51.90 | 40.30% - 63.50% |
| **diabetes duration** (n=77) | | | |
| Less than 3 years | 37 | 48.10 | 36.50% - 59.70% |
| More than 3 years | 40 | 51.90 | 40.30% - 63.50% |
| **Treatment follow** (n=77) | | | |
| Insulin | 6 | 7.80 | 2.90% - 16.20% |
| OGCA** | 71 | 92.20 | 83.80% - 97.10% |
| **Controlled T2D**[1] (n=77) | | | |
| Yes | 54 | 70.10 | 58.60% - 80.00% |
| No | 23 | 29.90 | 20.00% - 41.40% |
| **BMI*** ** | | | |
| More than 25Kg/m$^2$ | 56 | 72.70 | 61.40% - 82.30% |
| Less than 25Kg/m$^2$ | 21 | 27.30 | 17.70% - 38.60% |

* Type 2 diabetes. ** Oral Glucose Control Agents. Principally metformin here. *** Body Mass Index

[1]: Based on HbA1c levels
