# Supplementary material for: Is it a supplementary benefit to use anti-inflammatory agents in the treatment of type 2 diabetes?
Source: BMC Res Notes. 2017 Sep 8;10:471. doi: 10.1186/s13104-017-2785-4 (PMC5591512; doi:10.1186/s13104-017-2785-4)
Supplement: Supplementary file 5 — Additional file 5. Means of body mass index (BMI) and waist measurement in relation to sex. [file 13104_2017_2785_MOESM5_ESM.pdf]

Table S5: Means of body mass index (BMI) and waist measurement in relation to sex

	Frequency	Means	SD	P-value
**BMI**				
Male	46	27.15 Kg/m^2	3.82 Kg/m^2	
Female	31	29.02 Kg/m^2	4.14 Kg/m^2	**0.04***
**Waist measurement**				
Male	46	98.21 cm	12.22 cm	
Female	31	100.29 cm	11.32 cm	/
