# Supplementary material for: Is it a supplementary benefit to use anti-inflammatory agents in the treatment of type 2 diabetes?
Source: BMC Res Notes. 2017 Sep 8;10:471. doi: 10.1186/s13104-017-2785-4 (PMC5591512; doi:10.1186/s13104-017-2785-4)
Supplement: Supplementary file 6 — Additional file 6. Means of hs-CRP and HbA1c in relation to the sociodemographic and clinical features of participants. [file 13104_2017_2785_MOESM6_ESM.pdf]

Table S6: Means of hs-CRP and HbA1c in relation to the sociodemographic and clinical features of participants

	hs-CRP	P value	HbA1c (< 7%)[r]	P value
**Sex**				
Male	7.43 mg/l		6.46%	
Female	6.50 mg/l	0.66	6.22%	0.47
**Age**				
> 50 years	7.61 mg/l		6.24%	
< 50 years	4.77 mg/l	0.84	6.90%	0.10
**Diabetes duration**				
< 3 years	8.03 mg/l		6.44%	
> 3 years	6.15 mg/l	0.21	6.30%	0.65
**Treatment follow**				
OGCA	7.11 mg/l		6.38%	
Insulin	6.42 mg/l	0.89	6.18%	0.66
**BMI**				
< 25 Kg/m^2	7.36 mg/l		6.77%	
> 25 Kg/m^2	6.94 mg/l	0.89	6.22%	0.13
**Controlled T2D[1]**				
yes	5.34 mg/l		5.74%	
No	11.08 mg/l	**0.04***	7.98%	**0.00***
**Global physical activity**				
Weak	8.12 mg/l		6.46%	
Moderate	3.63 mg/l		6.49%	
Intense	5.98 mg/l		7.43%	
None	7.82 mg/l	0.33	5.94%	0.39

The values which we have represented in this table are the means of hs-CRP concentrations and HbA1c levels in each group

[r]: reference value

[1]: Based on HbA1c levels
