# Supplementary material for: Is it a supplementary benefit to use anti-inflammatory agents in the treatment of type 2 diabetes?
Source: BMC Res Notes. 2017 Sep 8;10:471. doi: 10.1186/s13104-017-2785-4 (PMC5591512; doi:10.1186/s13104-017-2785-4)
Supplement: Supplementary file 7 — Additional file 7. Means of hs-CRP and HbA1c in relation to feeding practices and physical activity level of participants. [file 13104_2017_2785_MOESM7_ESM.pdf]

**Table S7**: Means of hs-CRP and HbA1c in relation to feeding practices and physical activity level of participants

| | hs-CRP | P value | HbA1c (< 7%)[r] | P value |
|---|---|---|---|---|
| **Regular physical activity** | | | | |
| Yes | 4.58 mg/l | | 6.46% | |
| No | 8.40 mg/l | 0.39 | 6.32% | 0.68 |
| **Number of session per week** | | | | |
| Less than three | 6.18 mg/l | | 6.30% | |
| Three | 1.81 mg/l | | 6.21% | |
| More than three | 2.79 mg/l | 0.11 | 7.24% | 0.30 |
| **Time of each session** | | | | |
| 30 min | 4.54 mg/l | | 6.52% | |
| More than 30 min | 4.58 mg/l | 0.98 | 6.45% | 0.94 |
| **Number of meals per day** | | | | |
| Two meals | 4.93 mg/l | | 6.12% | |
| Three meals | 7.16 mg/l | | 6.40% | |
| Four meals | 16.36 mg/l | | 7.19% | |
| More than four meals | 7.31 mg/l | 0.37 | 5.99% | 0.75 |
| **Meals are taken morn, noon and afternoon** | | | | |
| Yes | 6.28 mg/l | | 6.41% | |
| No | 10.54 mg/l | 0.97 | 6.19% | 0.60 |
| **Gnaw between meals** | | | | |
| Yes | 7.41 mg/l | | 6.19% | |
| No | 6.75 mg/l | 0.37 | 6.52% | 0.31 |
| **Juice consumption** | | | | |
| Yes | 2.03 mg/l | | 6.33% | |
| No | 7.19 mg/l | 0.54 | 6.37% | 0.96 |
| **Alcohol consumption** | | | | |
| Yes | 6.01 mg/l | | 6.44% | |
| No | 7.76 mg/l | **0.05*** | 6.31% | 0.69 |
| **High blood pressure patient** | | | | |
| Yes | 5.79 mg/l | | 6.27% | |
| No | 7.67 mg/l | 0.93 | 6.41% | 0.68 |

The values which we have represented in this table are the means of hs-CRP concentrations and HbA1c levels in each group

[r]: reference value
