# Supplementary figures and images for: Is it a supplementary benefit to use anti-inflammatory agents in the treatment of type 2 diabetes?
Source: BMC Res Notes. 2017 Sep 8;10:471. doi: 10.1186/s13104-017-2785-4 (PMC5591512; doi:10.1186/s13104-017-2785-4)

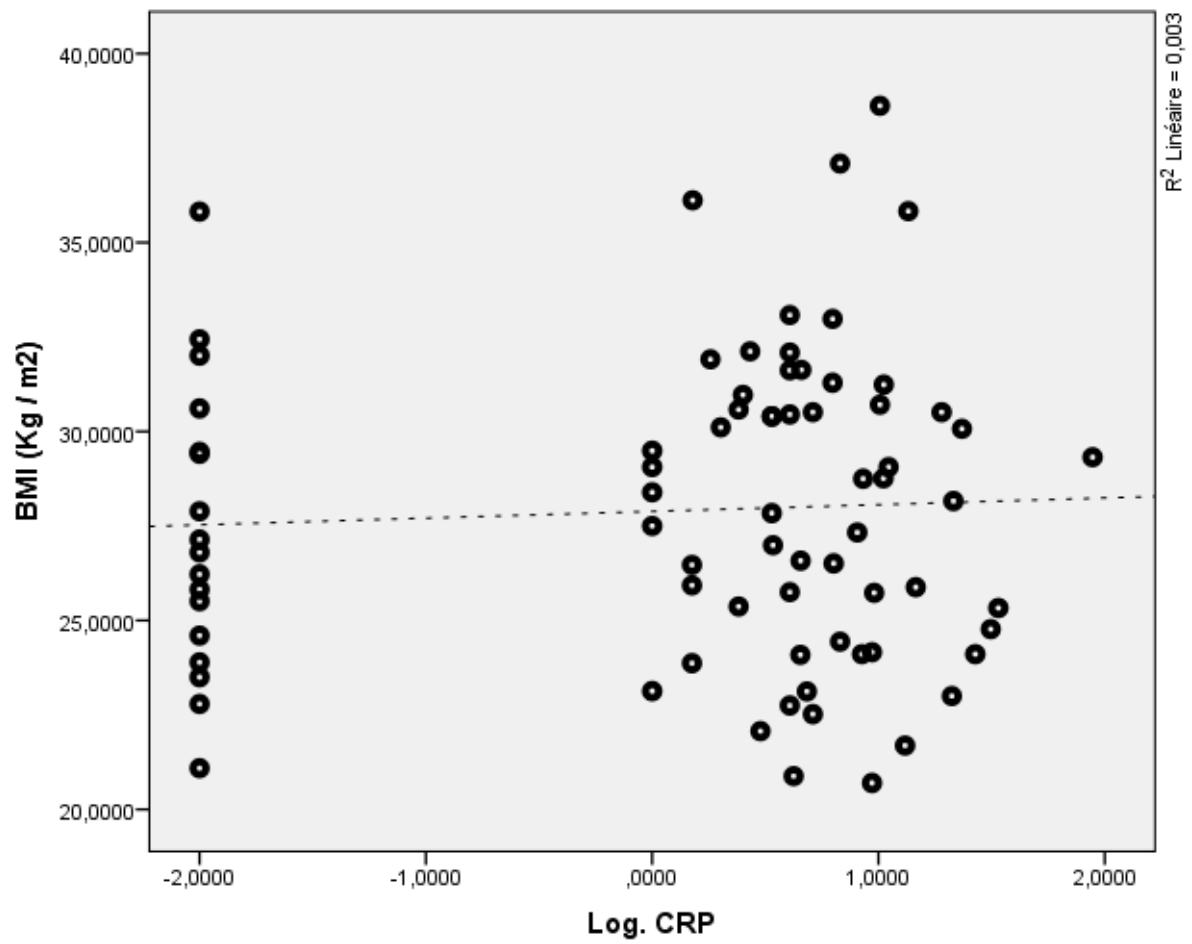

**Figure S1:** Dispersion of BMI and hs-CRP in the study population ( $r=0.053$ ; not significant)

Supplement: Supplementary file 8 — Additional file 8. Dispersion of BMI and hs-CRP in the study population. [file 13104_2017_2785_MOESM8_ESM.pdf]

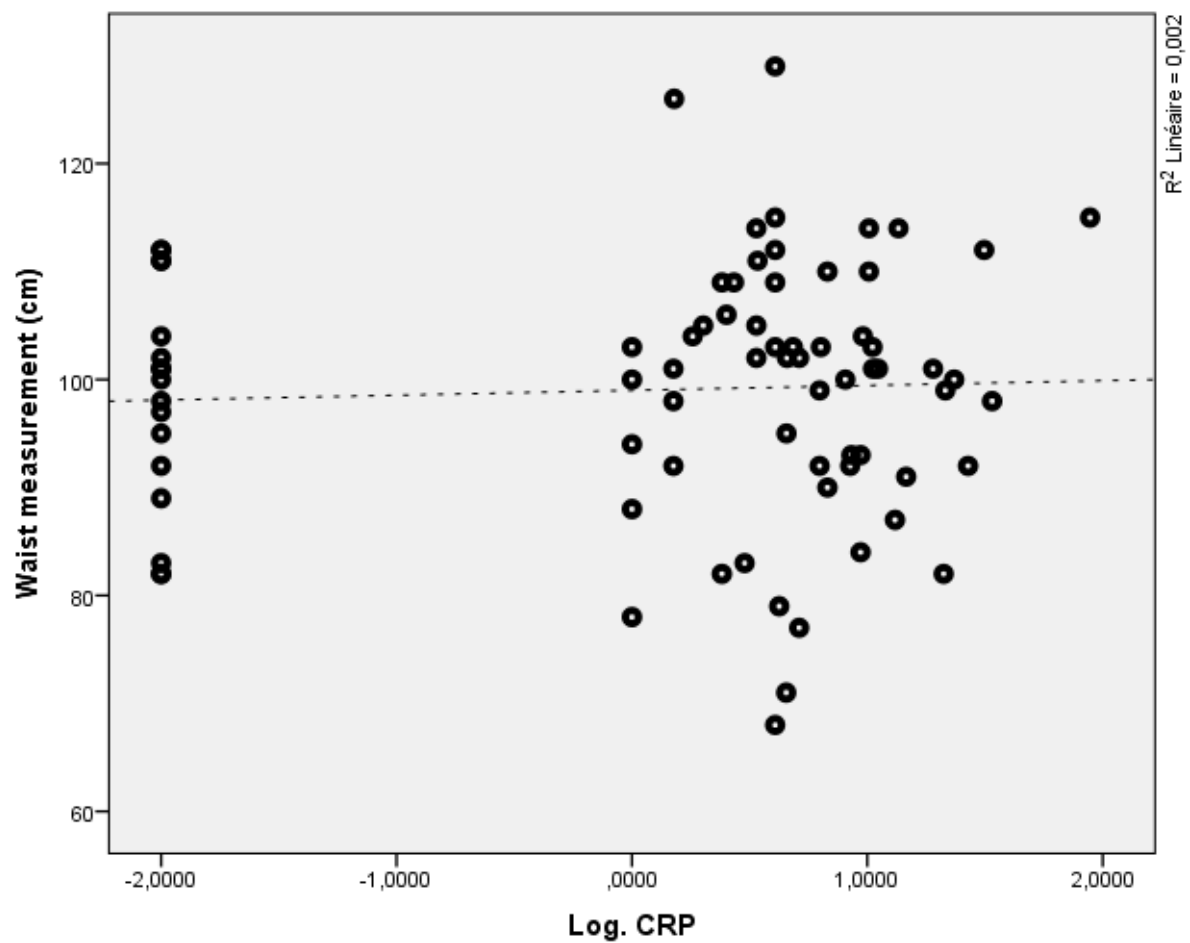

**Figure S2:** Dispersion of waist measurement and hs-CRP in the study population ( $r=0.046$ ; not significant)

Supplement: Supplementary file 9 — Additional file 9. Dispersion of waist measurement and hs-CRP in the study population. [file 13104_2017_2785_MOESM9_ESM.pdf]
