# Supplementary material for: Is it a supplementary benefit to use anti-inflammatory agents in the treatment of type 2 diabetes?
Source: BMC Res Notes. 2017 Sep 8;10:471. doi: 10.1186/s13104-017-2785-4 (PMC5591512; doi:10.1186/s13104-017-2785-4)
Supplement: Supplementary file 10 — Additional file 10. Dispersion of the daily doses of insulin used by patients on insulin-therapy and hs-CRP. [file 13104_2017_2785_MOESM10_ESM.pdf]

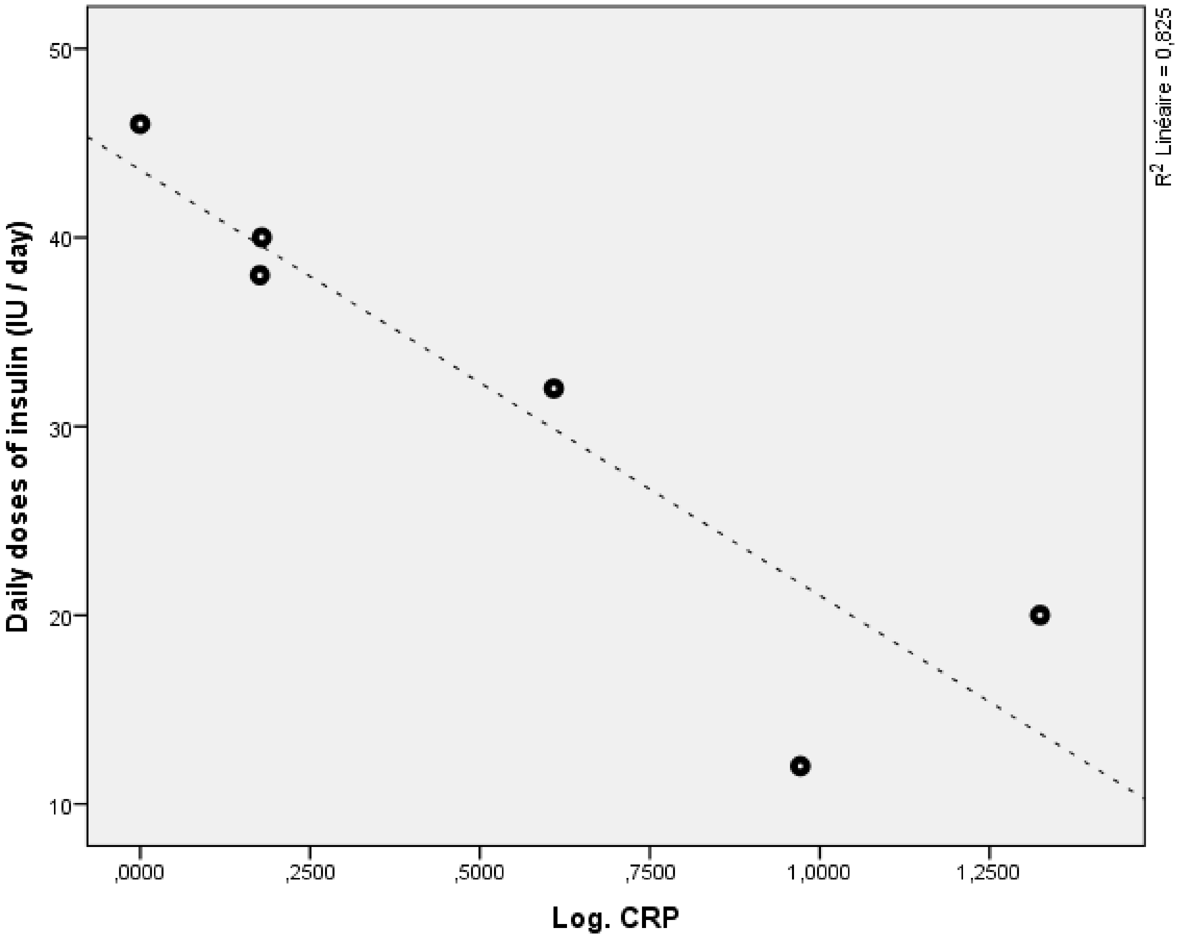

Figure S3: Dispersion of the daily doses of insulin used by patients on insulin-therapy and hs-CRP (r= - 0.908; P=0.012)
