# Supplementary material for: Is it a supplementary benefit to use anti-inflammatory agents in the treatment of type 2 diabetes?
Source: BMC Res Notes. 2017 Sep 8;10:471. doi: 10.1186/s13104-017-2785-4 (PMC5591512; doi:10.1186/s13104-017-2785-4)
Supplement: Supplementary file 11 — Additional file 11. Dispersion of the daily doses of oral glucose control agents used by the participants and hs-CRP. [file 13104_2017_2785_MOESM11_ESM.pdf]

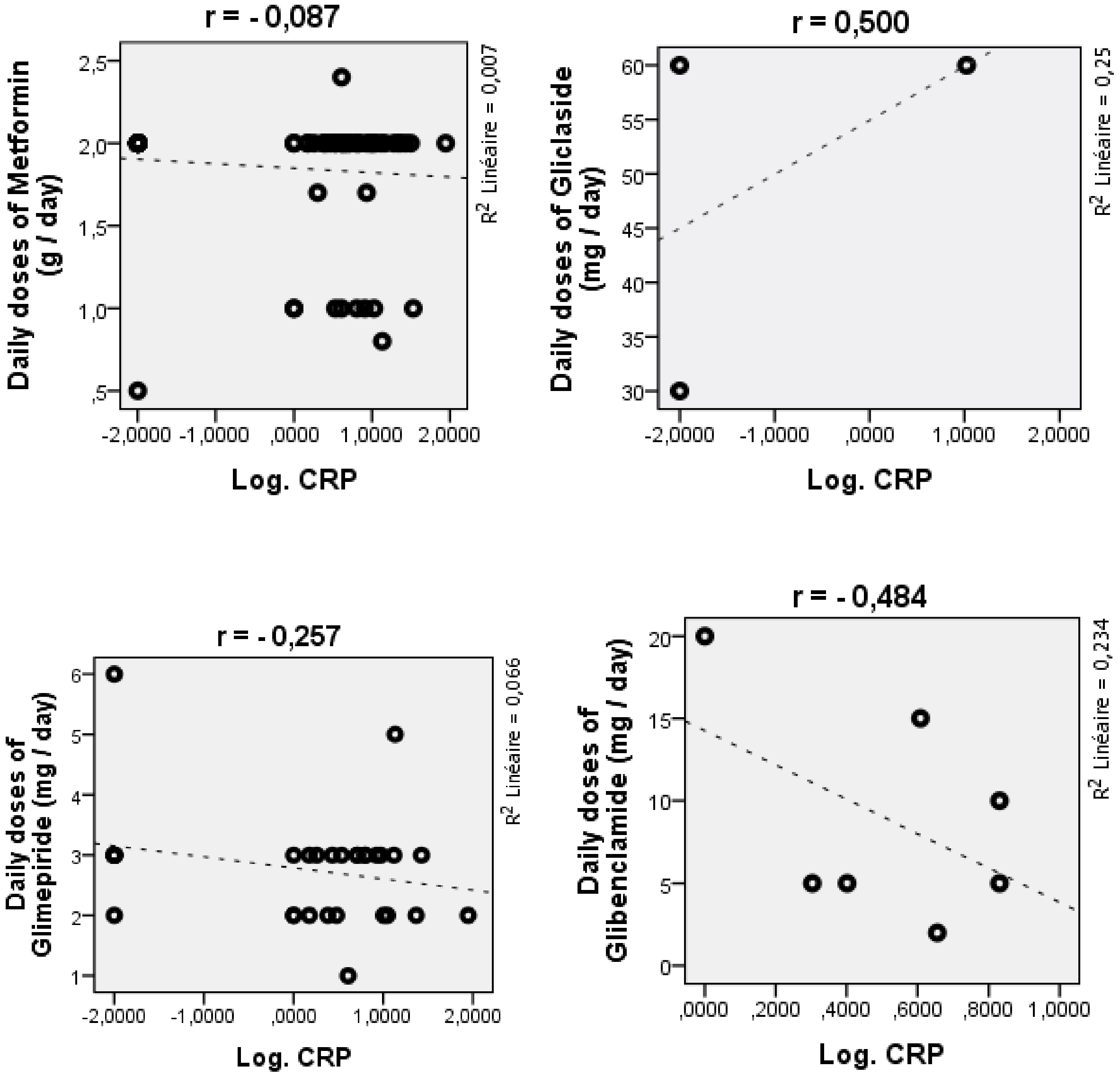

Figure S4: Dispersion of the daily doses of oral glucose control agents used by the participants and hs-CRP (r aren't significant)
