# Supplementary material for: Is it a supplementary benefit to use anti-inflammatory agents in the treatment of type 2 diabetes?
Source: BMC Res Notes. 2017 Sep 8;10:471. doi: 10.1186/s13104-017-2785-4 (PMC5591512; doi:10.1186/s13104-017-2785-4)
Supplement: Supplementary file 12 — Additional file 12. Dispersion of HbA1c and hs-CRP in well-controlled diabetes patients and in non-controlled diabetes patients. [file 13104_2017_2785_MOESM12_ESM.pdf]

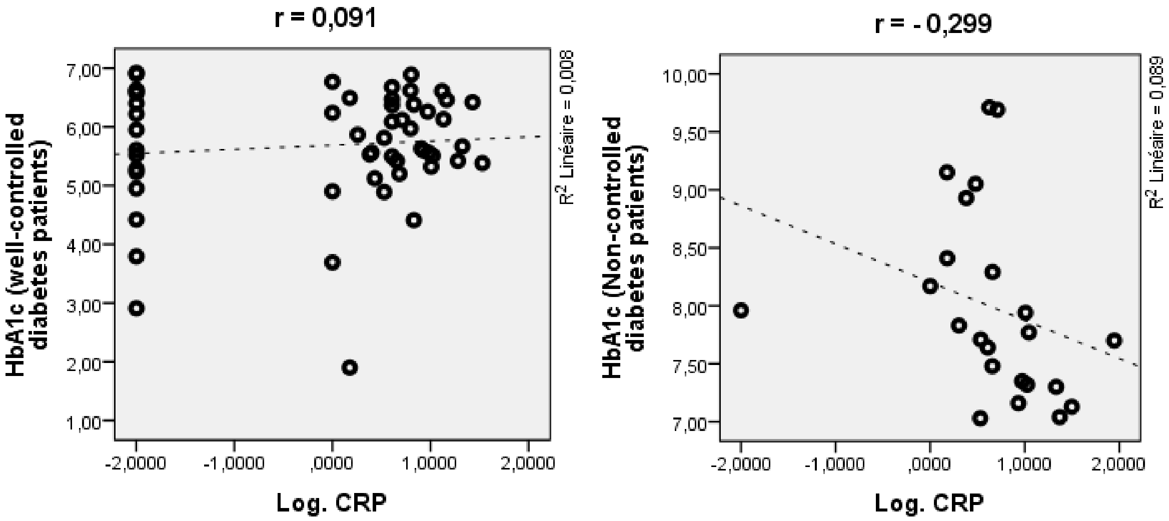

Figure S5: Dispersion of HbA1c and hs-CRP in well-controlled diabetes patients and in non-controlled diabetes patients (r aren't significant)
