# Supplementary material for: Is it a supplementary benefit to use anti-inflammatory agents in the treatment of type 2 diabetes?
Source: BMC Res Notes. 2017 Sep 8;10:471. doi: 10.1186/s13104-017-2785-4 (PMC5591512; doi:10.1186/s13104-017-2785-4)
Supplement: Supplementary file 13 — Additional file 13. Dispersion of diastolic arterial pressure and hs-CRP in the study population. [file 13104_2017_2785_MOESM13_ESM.pdf]

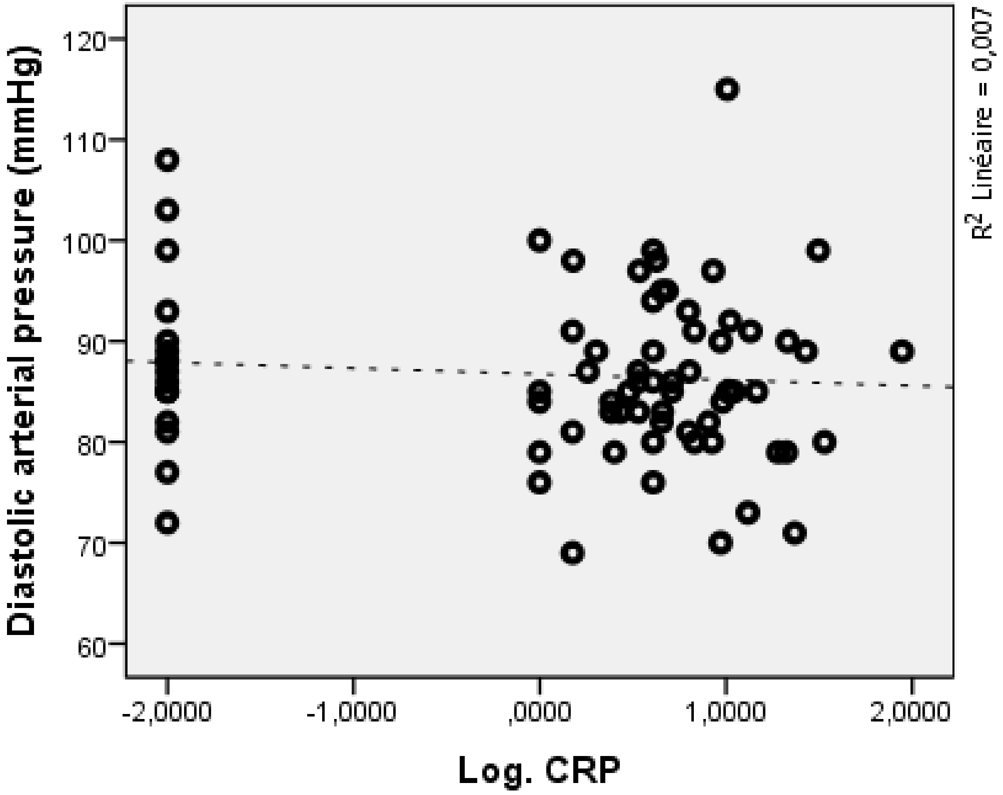

Figure S6: Dispersion of diastolic arterial pressure and hs-CRP in the study population (r= - 0.082; not significant)
